# Engineering a highly active thermophilic β-glucosidase to enhance its pH stability and saccharification performance

**DOI:** 10.1186/s13068-016-0560-8

**Published:** 2016-07-20

**Authors:** Wei Xia, Xinxin Xu, Lichun Qian, Pengjun Shi, Yingguo Bai, Huiying Luo, Rui Ma, Bin Yao

**Affiliations:** Key Laboratory for Feed Biotechnology of the Ministry of Agriculture, Feed Research Institute, Chinese Academy of Agricultural Sciences, No. 12 Zhongguancun South Street, Beijing, 100081 People’s Republic of China; College of Animal Science, Zhejiang University, Hangzhou, 310058 People’s Republic of China; Biotechnology Research Institute, Chinese Academy of Agricultural Sciences, Beijing, 100081 People’s Republic of China

**Keywords:** β-Glucosidase, *Talaromyce leycettanus*, Saccharification, pH stability, *O*-glycosylation, *Pichia pastoris*

## Abstract

**Background:**

β-Glucosidase is an important member of the biomass-degrading enzyme system, and plays vital roles in enzymatic saccharification for biofuels production. Candidates with high activity and great stability over high temperature and varied pHs are always preferred in industrial practice. To achieve cost-effective biomass conversion, exploring natural enzymes, developing high level expression systems and engineering superior mutants are effective approaches commonly used.

**Results:**

A newly identified β-glucosidase of GH3, Bgl3A, from *Talaromyces leycettanus* JCM12802, was overexpressed in yeast strain *Pichia pastoris* GS115, yielding a crude enzyme activity of 6000 U/ml in a 3 L fermentation tank. The purified enzyme exhibited outstanding enzymatic properties, including favorable temperature and pH optima (75 °C and pH 4.5), good thermostability (maintaining stable at 60 °C), and high catalytic performance (with a specific activity and catalytic efficiency of 905 U/mg and 9096/s/mM on *p*NPG, respectively). However, the narrow stability of Bgl3A at pH 4.0–5.0 would limit its industrial applications. Further site-directed mutagenesis indicated the role of excessive *O*-glycosylation in pH liability. By removing the potential *O*-glycosylation sites, two mutants showed improved pH stability over a broader pH range (3.0–10.0). Besides, with better stability under pH 5.0 and 50 °C compared with wild type Bgl3A, saccharification efficiency of mutant M1 was improved substantially cooperating with cellulase Celluclast 1.5L. And mutant M1 reached approximately equivalent saccharification performance to commercial β-glucosidase Novozyme 188 with identical β-glucosidase activity, suggesting its great prospect in biofuels production.

**Conclusions:**

In this study, we overexpressed a novel β-glucosidase Bgl3A with high specific activity and high catalytic efficiency in *P. pastoris.* We further proved the negative effect of excessive *O*-glycosylation on the pH stability of Bgl3A, and enhanced the pH stability by reducing the *O*-glycosylation. And the enhanced mutants showed much better application prospect with substantially improved saccharification efficiency on cellulosic materials.

**Electronic supplementary material:**

The online version of this article (doi:10.1186/s13068-016-0560-8) contains supplementary material, which is available to authorized users.

## Background

As one of the most abundant renewable energy sources on Earth, plant biomass mainly consists of lignocellulose, which is a complicated heterogeneous complex made up of hemicellulose, lignin and cellulose [1]. For the closest decades, developing efficient technologies to convert biomass materials into fuels has attracted focused attention of researchers [2, 3]. Moreover, the biodegradation of cellulosic materials has been reported to have potential importance in kinds of industrial and agricultural applications [4–7]. Some glycoside hydrolases (GHs) are the most effective enzymes to depolymerize cellulose. As generally known, endo-β-glucanase (EC 3.2.1.4, EG) that catalyzes the breakdown of internal β-1,4-linkages at random position of the glucose polymers, cellobiohydrolase (EC 3.2.1.91, CBH I and CBH II) that cuts off cellobiose residues from the reducing or nonreducing ends, and β-glucosidase (EC 3.2.1.21) that hydrolyzes single units from the nonreducing end into glucose [3, 8]. In detail, EGs catalyze the breakdown of internal β-1, 4-linkages at random position of the glucose polymer chain, while CBHs cut-off cellobiose residues from the ends (CBH I and CBH II cuts from the reducing and nonreducing ends, respectively). At the last step, generated cellobiose or cello-oligosaccharides are hydrolyzed into single units of glucose from the nonreducing end by β-glucosidases. And recent researches reveal that a class of enzymes now known as lytic polysaccharide monooxygenases (LPMOs) are also important for the decomposition of recalcitrant biological macromolecules such as plant cell wall and chitin polymers [9]. LPMOs cleave the chains at the surface of the crystalline polymer by oxidation of the polysaccharide chain to contribute to further enzymatic action and eventual degradation [10]. These enzymes were originally designated glycoside hydrolase family 61 and carbohydrate-binding module family 33, but are now classified as auxiliary activities 9 (formerly GH61), 10 (formerly CBM33) and 11 in the CAZy database [11].

Several cellulolytic GHs have been commercialized for industrial production of biofuels and chemicals [12–15]. For example, Celluclast 1.5L (Novo Nodisk A/S) from *Trichoderma reesei* ATCC 26921 and newly developed Cellic^®^ CTec2 and Cellic^®^ CTec3 are the most widely used commercial cellulolytic preparation [16, 17]. However, its low β-glucosidase activity makes supplementation of exogenous enzyme necessary for efficient biomass conversion [18, 19]. Since β-glucosidase plays a vital role in cellulose hydrolysis by undertaking the rate-limiting final step of hydrolyzing cellobiose, which is an intermediate product of cellulose hydrolysis and also a strong inhibitor of cellulase activities, into glucose [20, 21], it’s a common practice to supplement exogenous β-glucosidase to enhance the saccharification efficiency of cellulosic materials [22–24]. This challenge remains a major bottleneck in the bioconversion process, and recent research has, therefore, shown increased interest in the search for novel β-glucosidases. Based on the amino acid sequences, β-glucosidases have been classified into GH families 1, 3, 5, 9, 30 and 116. Although enzymes from different families and different organisms vary greatly in properties and functions, cost-effective production, high hydrolytic efficiency and great tolerance to unfavorable conditions are prerequisites of an industrial biocatalyst in various applications [25]. Of particular interest, β-glucosidases from thermophilic fungi are more favorable due to the high-temperature activity and good thermostability [26, 27]. Recent advances have been made in β-glucosidase engineering and production [28–30]; however, much work is yet to be addressed for the improvement of β-glucosidases for industrial applications.

Incompatibility between enzymatic properties and process conditions is a common issue for enzyme commercialization. Thus, to discover and modify enzymes with favorable properties is a research focus for efficient utilization of plant biomass [31, 32]. pH stability, as an essential enzymatic property, is closely related to the factors that affect protein structure, such as protein folding, interferential chemicals and posttranslational modifications (PTMs) [33]. PTMs occur on the amino acid side chains or termini by introducing new functional groups such as phosphate (phosphorylation), acetate (acylation), amide groups (amidation), methyl groups (methylation), or carbohydrate molecules (glycosylation) [34]. Many eukaryotic proteins also have attached to them in a process called glycosylation, which can promote protein folding and improve stability as well as serving regulatory functions. Glycosylation represents one major PTM of eukaryotic proteins, which occurs at asparagine of the consensus motif Asn-X-Thr/Ser (where X is any amino acid except Pro; *N*-glycosylation) or serine and threonine residues (*O*-glycosylation) [29]. In contrast to the absence of glycosylation in prokaryotic expression systems like *Escherichia coli*, glycosylation is common in eukaryotic host systems (i.e., *Pichia pastoris*) and play important roles in proper folding, transport, and stability of proteins [23, 34–38]. Yeast strains are widely used for recombinant protein production, during which both *O*- and *N*-glycosylations occur preceding protein secretion and have obvious effects on enzyme properties [39–41]. *P. pastoris* is capable of adding both *O*- and *N*-linked carbohydrate moieties to secreted proteins. In comparison with the intensive studies of *N*-glycosylation effect on enzyme properties [42], less was done to reveal the mechanism of *O*-glycosylation [43, 44].

The purpose of this study is to explore and develop a novel β-glucosidase with capacity of accelerating the conversion efficiency of cellulosic materials into glucose. We produced the β-glucosidase (Bgl3A) from *Talaromyces leycettanus* JCM12802 in *P. pastoris* and found its advantages and disadvantages for industrial applications. By removing *O*-glycosylation, we improved the pH stability of the enzyme and enhanced the saccharification efficiency of the enzyme combination with commercial cellulase.

## Results

### Gene cloning and sequence analysis

A gene fragment of ~4.3 kb containing the full-length β-glucosidase-encoding sequence (*bgl3A*, 2214 bp) was obtained from the genome of *T. leycettanus* JCM12802 using TAIL-PCR. *bgl3A* encodes a GH3 polypeptide of 737 amino acid residues and a termination codon. Deduced Bgl3A has a putative signal peptide at the N-terminus (residues 1–19) and a mature protein with the calculated molecular mass and *p*I value of 76.3 kDa and 4.98, respectively. Three potential *N*-glycosylation sites (Asn23, Asn207 and Asn278) and nine potential *O*-glycosylation sites (residues Ser313, Thr417, Thr418, Ser419, Thr 420, Thr421, Thr424, Thr425 and Ser429) were identified in deduced Bgl3A, indicating the possible abundant posttranslational modification of Bgl3A when expressed in eukaryotic system. Deduced Bgl3A exhibits the highest sequence identities of 83 % with a putative GH from *Neosartorya fischeri* NRRL 181 and 82 % with GH3 β-glucosidase from *Aspergillus fumigatus* var. RP-2014. Blast analysis against the PDB database indicates that Bgl3A shares a rather high identity of 73 % with a structure-resolved counterpart β-glucosidase, Cel3A, from *Hypocrea jecorina* (PDB: 3ZYZ) [45].

### Expression and purification of recombinant β-glucosidase Bgl3A

**Fig. 1:**
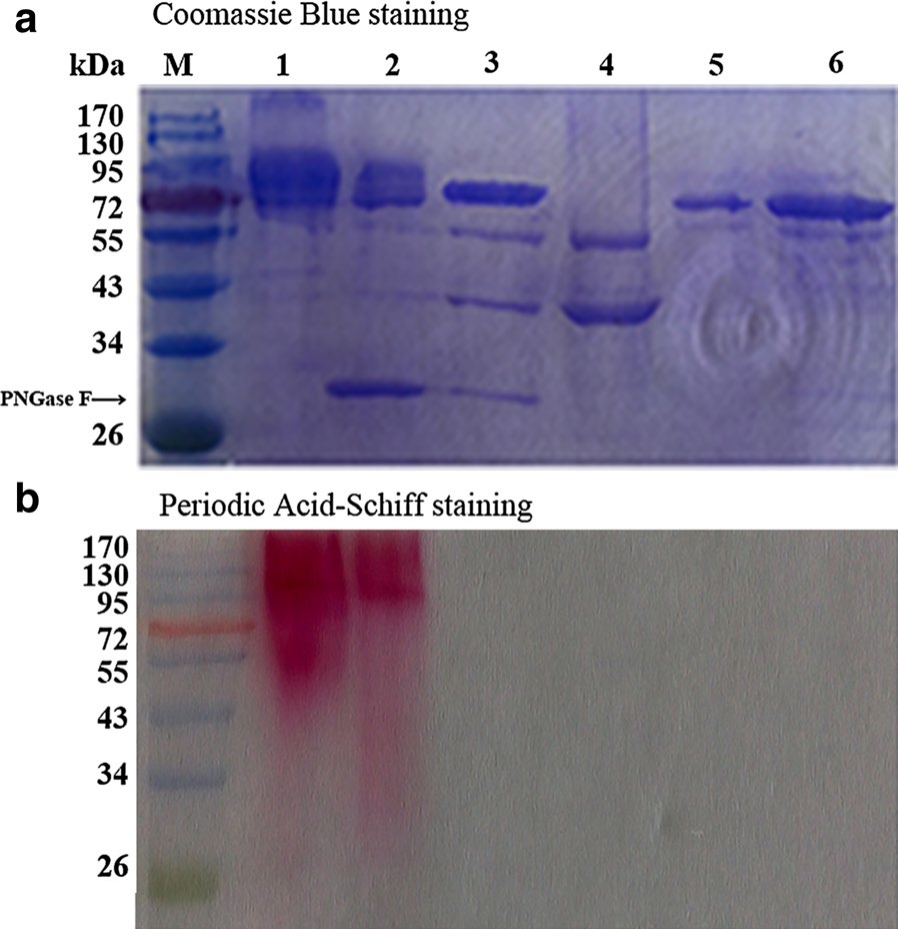
SDS-PAGE analysis of the purified recombinant β-glucosidase Bgl3A with and without deglycosylation treatment. **a** Coomassie Blue staining. *Lanes* M, the standard protein molecular weight markers; *1* the Bgl3A produced in *P. pastoris*; *2* the Bgl3A produced in *P. pastoris* and treated with PNGase F; *3* the Bgl3A produced in *P. pastoris* and treated with PNGase F followed by α-mannosidase; *4* the α-mannosidase; *5* the purified Bgl3A after sequential deglycosylation by PNGase F and α-mannosidase; *6* the Bgl3A produced in *E. coli*. **b** Periodic Acid-Schiff staining

The cDNA fragment of *bgl3A* without the signal peptide-coding sequence was successfully expressed in *P. pastoris* GS115 and *E. coli* BL21. The production level of crude Bgl3A in *P. pastoris* GS115 reached 6000 U/ml (approximate 6 g/L protein) in a 3 l fermentation tank after 132 h’ growth. The crude enzymes were then concentrated, desalted and purified. SDS-PAGE analyses indicated the purified Bgl3A expressed in *P. pastoris* spread over the gel with the apparent molecular masses of 76–100 kDa (Fig. 1a, lane 1), which was significantly higher than the calculated value (76.3 kDa). This significant molecular weight variance might be ascribed to the glycosylation occurred in *P. pastoris* during heterologous expression. In contrast, the purified recombinant Bgl3A produced in *E. coli* (*E. coli*-Bgl3A) migrated a single band of calculated value (Fig. 1a, lane 6), suggesting that no glycosylation occurred on *E. coli*-Bgl3A.

### Characterization of purified recombinant Bgl3A

**Fig. 2:**
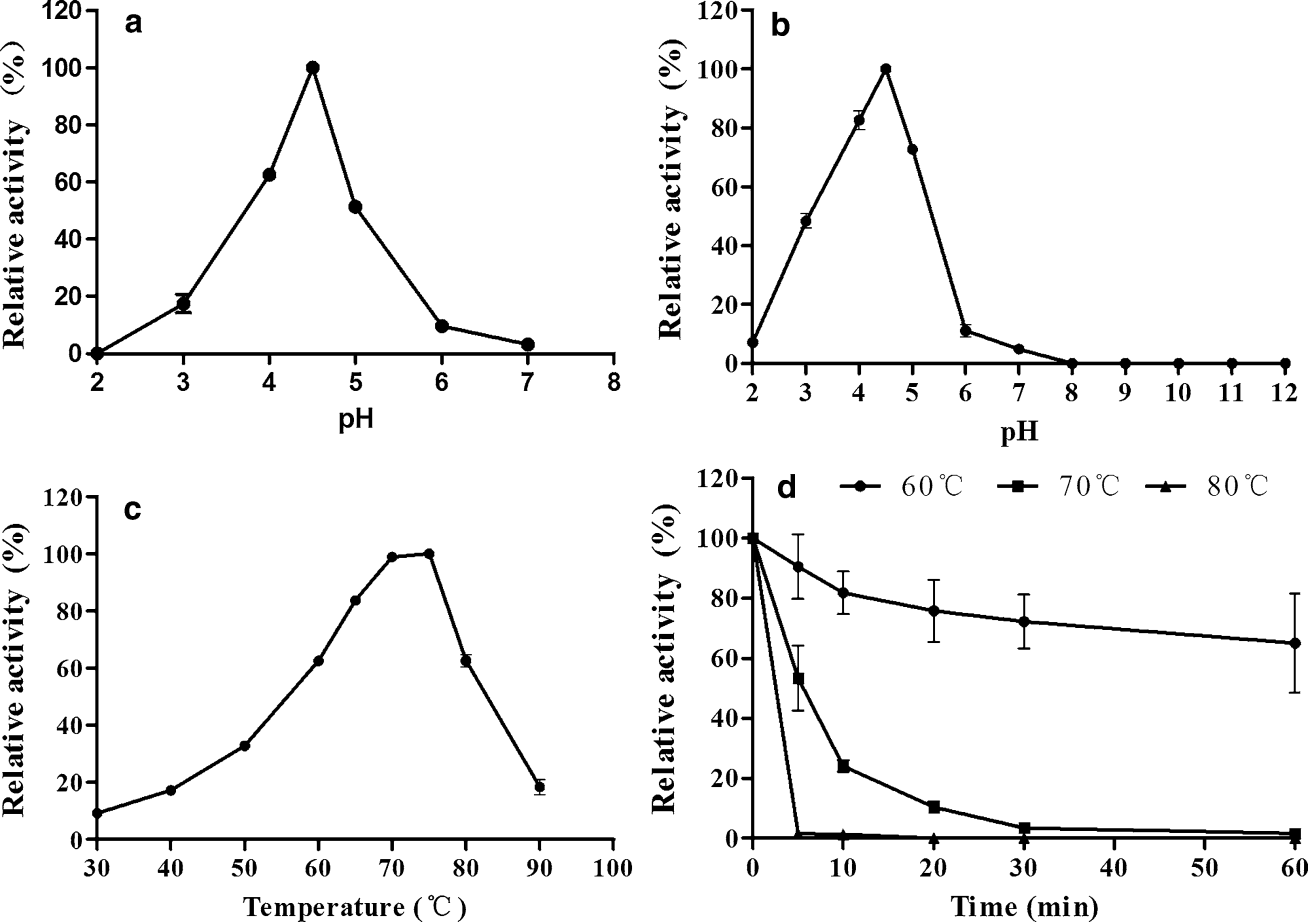
Enzymatic properties of the purified recombinant Bgl3A produced in *P. pastoris* using *p*NPG as the substrate. **a** Effect of pH on enzyme activities. **b** pH stability of Bgl3A after 1 h incubation at 37 °C. **c** Effect of temperature on enzyme activities. **d** Thermostability of Bgl3A at pH 4.5 and different temperatures up to 60 min. Each value in the panel represents the mean ± SD (*n* = 3)

**Fig. 3:**
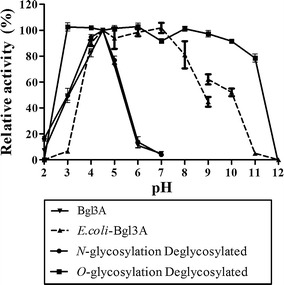
pH stabilities of glycosylated (produced in *P. pastoris*), non-glycosylated (produced in *E. coli*) and deglycosylated (treated with PNGase F and α-mannosidase) Bgl3A. The same amounts of enzymes were used for determination

The enzymatic properties of purified recombinant Bgl3A produced in *P. pastoris* were determined using 4-nitrophenyl β-d-glucopyranoside (*p*NPG) as the substrate. Purified Bgl3A was active over a narrow acid pH range, exhibiting optimal activity at pH 4.5 and retaining >65 % activity at pH 4.0–5.0 (Fig. 2a). The enzyme had poor pH stability, only retaining >70 % initial activity after 1 h incubation at pH 4.0–5.0 and 37 °C (Fig. 2b). When assayed Bgl3A activity at pH 4.5, it exhibited maximum activity at 75 °C and remained 65 % activity even at 80 °C (Fig. 2c). And Bgl3A was highly stable at 60 °C, retaining >65 % activity after 1 h incubation at 60 °C without substrate, but lost activity rapidly when treated at 70 and 80 °C (Fig. 2d). However, *E. coli*-Bgl3A had similar properties as that produced in *P. pastoris* (data not shown), but exhibited stability over a much broader pH range from 4.0 to 11.0 (Fig. 3, dash line).

The effects of metal ions and chemical reagents on the activity of Bgl3A were determined at the concentrations of 5 mM (Additional file 1). Bgl3A was highly resistant to most tested metal ions except for Ag^+^ and Cu^2+^ and remained active in the presence of chemical reagents EDTA, β-mercaptoethanol, and SDS. Moreover, Ca^2+^ enhanced the Bgl3A activity by 20 %. Similar result was observed for CfGlu1C from *Coptotermes formosanus* [46]. Ca^2+^ even has stimulatory effects on the TpeBlg3 stability at high temperatures, and 5 mM of Ca^2+^ enhances the activity by 58 % [47].

### Substrate specificity and kinetic parameters

The substrate specificities of purified recombinant Bgl3A are shown in Table 1. It showed high specific activities of 905 U/mg on *p*NPG and 265 U/mg on cellobiose. When using disaccharides of different linkages as the substrate, the enzymes showed different levels of preference, in the order of gentiobiose (β-1,6 linkage) >sophorose (β-1,2 linkage) >cellobiose (β-1,4 linkage). And for various aryl-glycoside substrates, Bgl3A was active with *p*NPG and amygdalin as preferred substrates.

**Table 1:** Substrate specificity of purified recombinant β-glucosidase Bgl3A

Substrate	Specific activity (U/mg)^a^
Disaccharides	
Gentiobiose (β-1,6)	393.2 ± 0.1
Sophorose (β-1,2)	342.8 ± 0.8
Cellobiose (β-1,4)	265.5 ± 0.2
Aryl-glycosides	
*p*-Nitrophenyl β-d-glucopyranoside	905.0 ± 0.1
*p*-Nitrophenyl β-d-cellobioside	76.6 ± 0.0
Amygdalin	377.4 ± 0.2
Genistin	175.3 ± 0.3
Daidzin	154.6 ± 0.9
Glycitin	75.6 ± 0.6

^a^Data is shown as mean ± standard deviation (*n* = 3)

The kinetics of Bgl3A on substrates *p*NPG and cellobiose are shown in Table 2. Bgl3A exhibited much higher substrate affinity (*K*_m_, 57-folds) and catalytic efficiency (*k*_cat_/*K*_m_, 120-folds) on *p*NPG than on cellobiose. The glucose inhibition constant *K*_i_ value of Bgl3A was determined to be 14 mM.

**Table 2:** Kinetic parameters of β-glucosidase Bgl3A and its two mutants

Substrate	Enzyme	Specific activity (U/mg)	*K*_i_ (mM)	*K*_m_ (mM)	*V*_max_ (U/mg)	*k*_cat_ (/s)	*k*_cat_/*K*_m_ (/s/mM)
*p*NPG	Bgl3A	905	14.0	0.18	1309	1664	9096
	M1	1019	17.0	0.31	1408	1791	5778
	M2	843	12.3	0.36	1679	2136	4664
Cellobiose	Bgl3A	265.5	ND	10.4	618.4	786.0	75.8
	M1	209.4	ND	8.4	531.6	676.0	80.5
	M2	197.6	ND	9.3	733.0	932.6	100.3

*ND* not determined

### Deglycosylation and determination of pH stability of deglycosylated enzymes

Results above, illustrated that Bgl3A is a highly active, thermophilic, acidic β-glucosidase, which is of much value for various industrial applications. The only defect is its poor pH stability. As we all know, prokaryotic expression systems differ hugely from eukaryotic systems in several aspects such as codon preference, secretion pathways and posttranslational modification. Among them, posttranslational modification influences most on the structure and property of the expressed protein. And *N*-glycosylation and *O*-glycosylation take over the largest part of posttranslational modification in yeast cells. The great pH stability difference between Bgl3A and *E. coli*-Bgl3A might be ascribed to glycosylation. Thus the *N*- and *O*-glycans of Bgl3A were removed by PNGase F and α-mannosidase sequentially, and determination of the pH stability of deglycosylated enzymes were done to verify whether or not glycosylation affected the pH tolerance of Bgl3A expressed in *P. pastoris*. The changes of molecular weight after deglycosylation were determined by SDS–PAGE (Fig. 1a). According to the result, *N*-deglycosylated Bgl3A showed lower coomassie brilliant blue staining at the area of larger molecular weight (lane 2), indicating that PNGase F effectively eliminated partial glycans, i.e., *N*-glycans; further *O*-deglycosylation obtained a single brand (lane 5) which migrated to nearly the same distance with *E. coli*-Bgl3A (lane 6). Periodic acid-schiff (PAS) staining was also used to identify glycoprotein (Fig. 1b). Both Bgl3A and *N*-deglycosylated Bgl3A were dyed pinkish red, while *N*- and *O*-deglycosylated Bgl3A and *E. coli*-Bgl3A were not. It indicated that Bgl3A has both *N*- and *O*-glycosylations, which are successfully eliminated by PNGase F and α-mannosidase sequentially.

The pH stabilities of deglycosylated enzymes were compared with that of Bgl3A and *E. coli*-Bgl3A (Fig. 3). The pH stability of *N*-glycosylated Bgl3A had no difference from that of Bgl3A, while *O*- and *N*-glycosylated Bgl3A exhibited much better pH tolerance, retaining stable over pH 3.0–11.0. It revealed the influence of *O*-glycosylation on pH stability of Bgl3A.

### The stability and kinetic parameters of *O*-glycosylation sites mutants

**Fig. 4:**
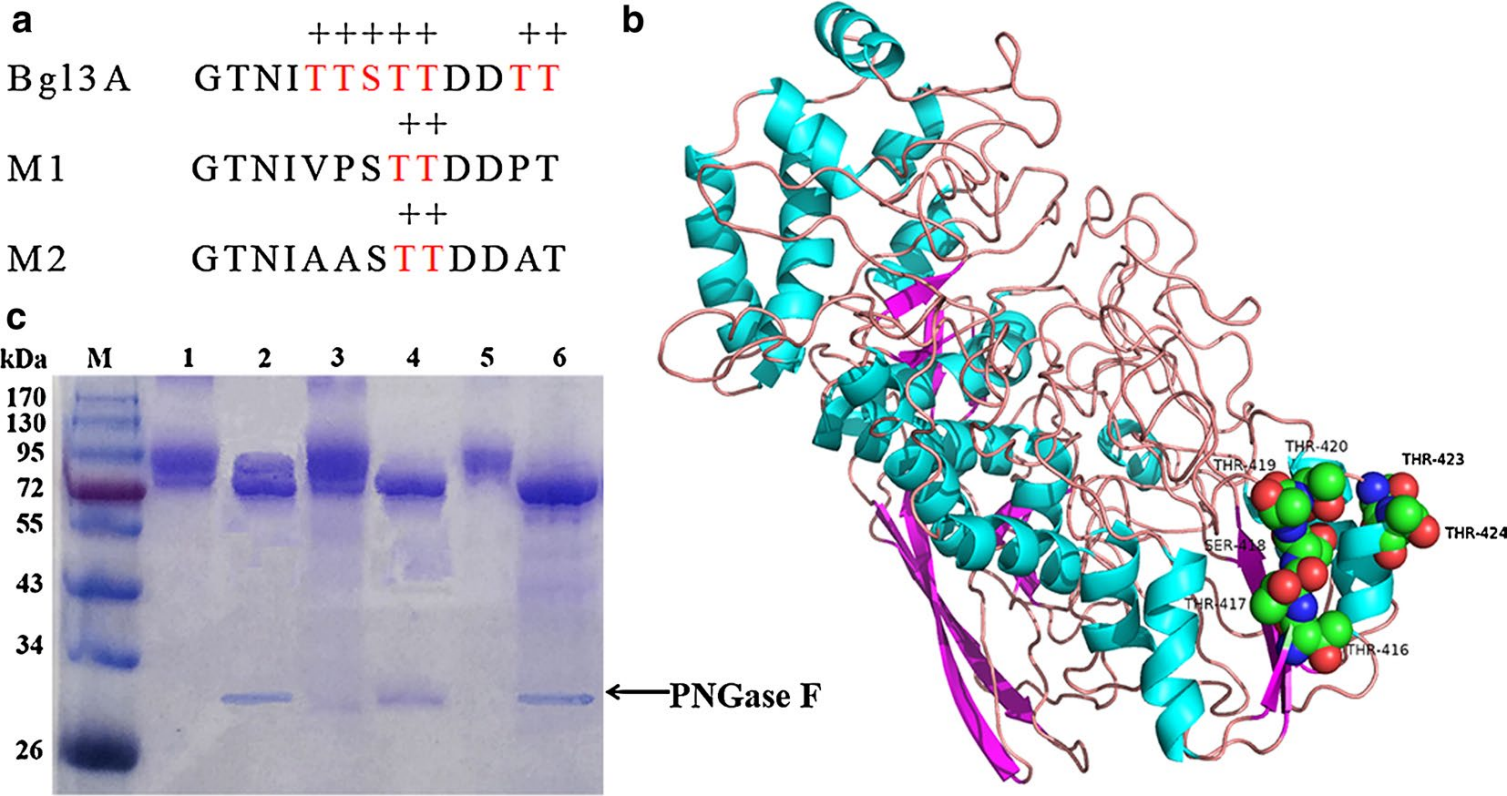
Sequence, structure and molecular mass analyses of wild type and mutant Bgl3A proteins. **a** The amino acid sequences of wild type and mutant enzymes with potential *O*-glycosylation sites (http://www.cbs.dtu.dk/services/NetOGlyc/) indicated. **b** Modeled Bgl3A with Cel3A of *H. jecorina* (PDB: 3ZYZ) as the template. The potential glycosylated residues are shown in balls. **c** SDS–PAGE analysis of the wild type and mutant enzymes produced in *P. pastoris* and treated with/without PNGase F. *Lanes* M, the standard protein molecular weight markers; *1* the purified Bgl3A; *2* the *N*-deglycosylated Bgl3A; *3* the purified M1; *4* the *N*-deglycosylated M1; *5* the purified M2; *6* the *N*-deglycosylated M2

**Fig. 5:**
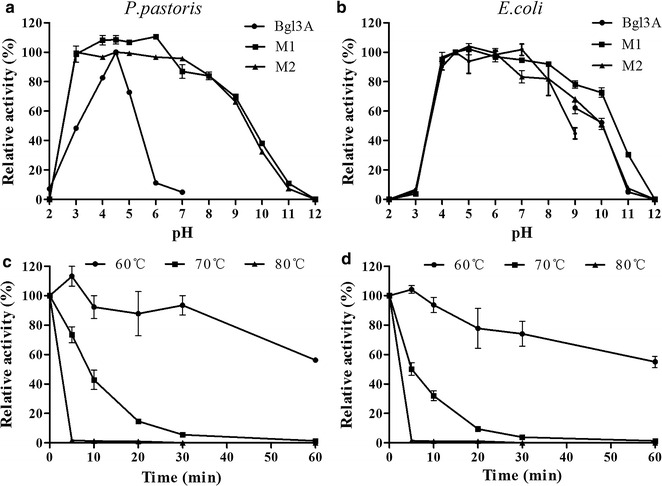
pH stabilities of wild type and mutant Bgl3A at the same amounts. **a** Enzymes expressed in *P. pastoris* GS115; **b** Enzymes expressed in *E. coli* BL21; **c** Thermostability of mutant M1 at 60, 70, and 80 °C up to 60 min; **d** Thermostability of mutant M2 at 60, 70, and 80 °C up to 60 min

As shown above, *O*-glycosylation was predicted to mainly occur at residues 417–429 of Bgl3A. In contrast, its close homolog, the GH3 β-glucosidase NfBGL1 from *N. fischeri* P1 [48] (73 % identity), is much more pH tolerant and has less potential *O*-glycosylation sites at this location. Thus two Bgl3A mutants, designated M1 and M2, with sequence substitution from NfBGL1 and *O*-glycosylation site substitution by alanine (Fig. 4a, b), were constructed to determine the influence of *O*-glycosylation on pH stability. Both mutants were expressed in *P. pastoris* and *E. coli*, respectively. SDS–PAGE analysis (Fig. 4c) indicated that *N*-glycans were effectively eliminated from wild type and mutant proteins by PNGase F. As results, *N*-deglycosylated Bgl3A appeared as a dispersing band in the gel since it still contained *O*-glycosylation, while two mutants migrated single bands with theoretical molecular masses. Moreover, the enzyme pH stability at 37 °C varied a lot when produced in different expression systems (Fig. 5a, b). Mutants M1 and M2 expressed in *P. pastoris* showed tolerance over a broader pH range than wild type Bgl3A (Fig. 5a), and had no obvious variation in thermostability (Fig. 5c, d). In contrast, the wild type and mutant enzymes expressed in *E. coli* had no significant difference in pH stability, retaining stable over a pH range of 4.0–10.0.

In comparison with wild type Bgl3A, mutants M1 and M2 had no much changes in specific activity, inhibition constant and kinetics, although their catalytic efficiencies towards *p*NPG were lowered significantly because of increased *K*_m_ values (Table 2). Analysis of the secondary structures of all recombinant proteins by CD spectrometry indicated little variance among proteins expressed in the same host (Additional file 2). The results suggested that residue substitution had no effect on the folding structure of Bgl3A, while proteins produced by different hosts had slight difference.

### Enzymatic saccharification of cellulose materials

**Fig. 6:**
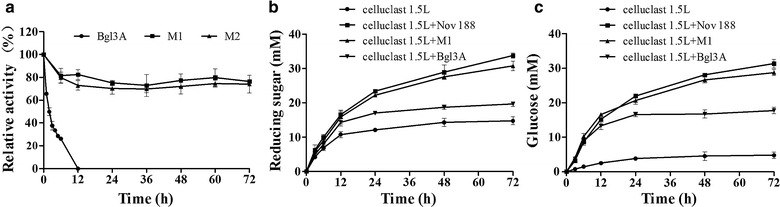
Enzyme stability and saccharification efficiency of wild type and mutant Bgl3A (10 U/g dry material) in combination with commercial cellulase (5 U/g dry material). The pretreated corn stover was used as the substrate. **a** Enzyme stability under pH 5.0 and 50 °C; **b** The reducing sugar released by enzyme(s); **c** The glucose released by enzyme(s)

The commercial cellulase preparation Celluclast 1.5L (Novo Nodisk A/S) from the hyperproducing mutant strain *Trichoderma reesei* ATCC 26921, which is most commonly used in biomass degradation, works best at pH 5.0 and 50 °C. So, to verify the application values of wild type Bgl3A and two modified mutants under factual conditions, the stability of enzymes was determined at pH 5.0 and 50 °C (Fig. 6a). The two mutants kept active up to 72 h, remaining over 70 % relative maximum activity, while wild type Bgl3A had a continuous declination in activity. The preferential order of stability was M1>M2>wild type Bgl3A.

Application potentials of M1 and wild type Bgl3A in saccharification of cellulosic materials were compared with that of commercial Novozyme 188 (Sigma-Aldrich). As shown in Fig. 6b and c, the saccharification efficiencies of Bgl3A and preferable mutant M1 were compared with commercial Novozyme 188 (Sigma-Aldrich) using corn stover as the cellulosic materials. In the blank control group, the commercial *T. reesei* cellulase Celluclast 1.5L (5 FPU (filter paper activity)/g dry material) released 14.78 mM of reducing sugars from corn stover over 72 h incubation at pH 5.0 and 50 °C, in which glucose accounted for 4.79 (32.4 %). When added β-glucosidase at the dosage of 10 BGU (β-glucosidase activity)/g dry material, wild type Bgl3A, mutant M1 and commercial Novozyme 188 showed different synergistic actions to promote the saccharification. By releasing 33.83 mM of reducing sugars and 31.35 mM glucose (the glucose conversion rate is 92.7 %), the synergistic action of Celluclast 1.5L and Novozyme 188 achieved a twofold increase in saccharification efficiency. In contrast, wild type Bgl3A with desirable catalytic properties in combination with Celluclast 1.5L only released 19.64 mM of reducing sugars and 17.75 mM of glucose, which are only 58.0 and 56.6 % of that of Celluclast 1.5L and Novozyme 188. However, mutant M1 showed improved performance in synergistic enzymatic saccharification. The yields of reducing sugars and fermentable glucose by Celluclast 1.5L and mutant M1 were increased substantially to 30.79 and 28.7 mM, respectively, which were both 1.6-fold of that of wild type Bgl3A. The glucose conversion rate of mutant M1 was 93.2 %, which was higher than that of Novozyme 188.

## Discussion

Thermophilic fungi are well-known to produce abundant plant cell wall-degradating hydrolases with superior characters, such as great catalytic performance, high expression level and excellent stability [16, 20, 21, 24, 49–51]. In decades, studies have been focused on heterologous expression of fungal β-glucosidases in *P. pastoris* [52–55] and engineering of β-glucosidases for property improvement [31, 56, 57]. In this study, a novel GH3 β-glucosidase, Bgl3A, was identified from thermophilic *T. leycettanus* JCM12802, and expressed in *P. pastoris* GS115 at high level. The determinations on enzymatic characteristics showed that Bgl3A was a thermophilic and acidic β-glucosidase with good thermostability and high-level production. The optimal temperature (75 °C) of Bgl3A was higher than that of most fungal β-glucosidases (45–65 °C) [51, 55, 58–60] including the most widely used commercial Novozyme 188 [21]. And its high specific activity and catalytic efficiency towards *p*NPG and cellobiose are much greater than most β-glucosidases [51, 54, 60]. These advantages make Bgl3A one of the most qualified candidates for industrial applications.

Moreover, the application bottleneck of Bgl3A, i.e., the narrow pH stability range (only pH 4.0–5.0 when expressed in *P. pastoris*), has been overcome by site-directed mutagenesis. Enzyme adaptation and tolerance to changeable conditions is one of the most important properties for industrial applications. There was no doubt that the defect in pH stability would limit the performance of the enzyme in practical uses. So it was of great importance to find out the reason that caused the instability and even to improve the enzyme by molecular modification. Glycosylation is known to be the main PTM of secretory proteins in *P. pastoris*. As previously mentioned, glycosylation has diverse effects on enzyme properties. Sometimes the effect is unexpected and unfavorable. In the case of Bgl3A that contains three potential *N*-glycosylation sites (Asn23, Asn207 and Asn278) and nine *O*-glycosylation sites (residues 313, 417–421, 424, 425 and 429), the enzyme was heavily glycosylated when expressed in *P. pastoris*, and was liable at pHs lower than 4.0 or higher than 5.0. However, when expressed in *E. coli*, *E. coli*-Bgl3A without any glycosylation was stable over a broad range of pH 4.0–10.0. It could be inferred that the variance of PTMs probably changed the recombinant proteins. Through the sequential enzymatic deglycosylation of *N*-linked and *O*-linked glycans, we found that it is *O*-glycosylation that accounts for the pH liability of Bgl3A. By removing two potential *O*-glycosylation sites, both mutants M1 and M2 gained pH tolerance over a much broader pH range (3.0–10.0) without unfavorable side-effects on other enzymatic and catalytic properties. Moreover, it was also found that *O*-deglycosylation by α-mannosidase and removal of *O*-glycosylation sites by site-directed mutagenesis both improved the enzyme pH stability at similar levels, while had no effect on mutant thermostability. The results confirmed that *O*-glycosylation is the main reason accounting for the narrowed pH stability range. As a whole, we concluded that excessive *O*-glycosylation could negatively affect the pH stability of β-glucosidase Bgl3A, and reducing the degree of *O*-glycosylation could dramatically improve the pH tolerance of the enzyme. Considering the far distance of *O*-glycosylation sites from the catalytic pocket of Bgl3A, *O*-glycans may influence Bgl3A stability by changing the enzyme spatial structure.

As known, the conversion of polysaccharides to glucose during saccharification process is regarded as both the rate and cost limiting steps in the production of biofuels efficient utilization of cellulose biomass. Therefore, the performance of a β-glucosidase in the process of synergetic enzymatic saccharification is undoubtedly a symbolic index to estimate its application value. With better stability and unchanged activities, mutant M1 released 1.6-folds fermentable sugars compared to wild type Bgl3A, which was approximately equal to that of commercial β-glucosidase preparation Novozyme 188 with the same activity units in the continuous saccharification process of 72 h under pH 5.0 and 50 °C. Moreover, mutant M1 had higher catalytic efficiency (80.5/s/mM) than commercial Novozyme 188 (36/s/mM) [21] towards natural substrate cellobiose, and produced comparable glucose yield as Novozyme 188. In other words, mutant M1 was more cost-effective since it needed less mass of enzyme protein to reach the required activity. Factually, data also showed that the concentration of glucose released by mutant M1 at the time point of 12 h was 16.61 mM, which was close to the *K*_i_ value of 17.0 mM. It illustrated that mutant M1 had better performance on cellobiose hydrolyzing at low glucose concentration. And the overtaking of Novozyme 188 during later period might be benefit from its rather high product inhibition constant (*K*_i_ value of 56.0 mM glucose) [22].

## Conclusions

β-Glucosidase plays a rate-limiting role in biomass conversion. In this study, a thermophilic, acidic, thermostable β-glucosidase from thermophilic *T. leycettanus* JCM12802 was identified and expressed in *P. pastoris* at high level. To meet the requirements of practical applications, the enzyme was further improved for pH stability over a broader range. As results, the mutant retained pH stability over 3.0–11.0 and exhibited comparable performance to commercial enzyme in saccharification of cellulosic materials. This study, not only reveals the negative effect of *O*-glycosylation on pH stability, but also provides an excellent enzyme candidate for efficient biomass conversion.

## Methods

### Strains, media, vectors and chemicals

The filamentous fungus *T. leycettanus* JCM12802 was preserved in our laboratory and routinely cultured in the wheat bran medium containing 5 g/l NaCl, 5 g/l (NH_4_)_2_SO_4_, 1 g/l KH_2_PO_4_, 0.5 g/l MgSO_4_·7H_2_O, 0.2 g/l CaCl_2_, 0.01 g/l FeSO_4_·7H2O, 10 g/l agar and 24 g/l wheat bran at 45 °C for 6 days. *Escherichia coli* Trans1-T1 and vector pEASY-T3 (TransGen, Beijing, China) were used for gene cloning. Vectors pET30a(+) and pPIC9 and *E. coli* BL21(DE3) and *P. pastoris* GS115 (Invitrogen, Carlsbad, CA) were used for prokaryotic and eukaryotic expression. The DNA purification kit, restriction endonucleases and *LATaq* DNA polymerase were purchased from TaKaRa (Otsu, Japan). T4 DNA ligase and the total RNA isolation system kit were purchased from Promega (Madison, WI). The cDNA synthesis kit was purchased from TransGen.

Barley β-glucan, Avicel, 4-nitrophenyl β-d-glucopyranoside (*p*NPG), 4-nitrophenyl β-d-xylopyranoside (*p*NPX), 4-nitrophenyl α-l-arabinofuranoside (*p*NPAf), 4-nitrophenyl α-d-galactopyranoside (*p*NPGal), 4-nitrophenyl α-l-arabinopyranoside (*p*NPAb), 4-nitrophenyl β-d-cellobioside (*p*NPC), disaccharides cellobiose, sophorose and gentiobiose, and soybean flavones daidzin, genistin and glycitin were purchased from Sigma-Aldrich (St. Louis, MO). Sodium carboxymethylcellulose (CMC-Na), laminarin and lichenin were obtained from Megazyme (Wicklow, Ireland). All other chemicals were of analytical grade and commercially available.

### Gene cloning and sequence analysis

Genomic DNA of strain *T. leycettanus* JCM12802 was extracted and purified as the template. To obtain the core region of the β-glucosidase gene (*bgl3A*), a degenerate primer set specific for fungal GH3 β-glucosidases (DP-F and DP-R, shown in Additional file 3) was designed based on the conserved amino acid sequences SSNIDD and GLDMT(A)MPGD(S). The resulting PCR product was gel purified, ligated with pEasy-T3 cloning vector and then transformed into *E*. *coli* Trans1-T1 for sequencing. The flanking regions were obtained by TAIL-PCR [61] using a genome walking kit (TaKaRa) and three pairs of specific nested primers (shown in Additional file 1: Table S1), sequenced, and assembled with the known core sequence to give the full-length *bgl3A*.

The total RNA of *T. leycettanus* JCM12802 was extracted from the mycelia after 4 days’ growth in the inducing medium, and was reverse transcribed into cDNA by TransScript^®^ One-Step gDNA Removal and cDNA Synthesis SuperMix kit (TransGen). The cDNA fragment of *bgl3A* without the signal peptide-coding sequence was then amplified with an annealing temperature of 60 °C and two primer sets (shown in Additional file 1: Table S1) specific for prokaryotic and eukaryotic expression. The PCR products were purified and ligated into the pEASY-T3 vector for sequencing.

Nucleotide and protein sequences were aligned using the BLASTn and BLASTp programs (http://www.ncbi.nlm.nih.gov/BLAST/), respectively. Vector NTI Advance 10.0 software (Invitrogen) was used to analyze the nucleotide sequence and to predict the molecular weight and *p*I of deduced proteins. Genes, introns, exons and transcription initiation sites were predicted using the online software FGENESH (http://linux1.softberry.com/berry.phtml). Multiple sequence alignments were performed with the ClustalW software. Putative signal peptide and glycosylation sites were predicted by the SignalP 4.1 server (http://www.cbs.dtu.dk/services/SignalP/) and the NetNGlyc 1.0 Server (http://www.cbs.dtu.dk/services/NetNGlyc/), respectively.

### Enzyme expression and purification in *P. pastoris*

The cDNA fragment without the signal peptide-coding sequence and the pPIC9 vector were both digested by *EcoR*I and *Not*I and ligated into in-frame fusion of the α-factor signal peptide to construct the recombinant plasmids, which were linearized using *Bgl*II and transformed into *P. pastoris* GS115 competent cells by electroporation using a Gene Pulser X cell Electroporation System (Bio-Rad, Hercules, CA). Minimal dextrose medium (MD) plates were used to screen positive transformants. The positive transformants were transferred to buffered glycerol complex medium (BMGY) and grown at 30 °C for 2 days. The cells were collected by centrifugation and resuspended in buffered methanol complex medium (BMMY) containing 0.5 % methanol for induction. The β-glucosidase activities of the culture supernatants were assayed using *p*NPG as the substrate, and the transformants exhibiting the highest β-glucosidase activities were subjected to high level expression in 1-l Erlenmeyer flasks and 3-l fermentor according to the *Pichia* expression manual (Invitrogen).

The culture supernatants aforementioned were collected by centrifugation (12,000×*g*, 4 °C, and 10 min) to remove cell debris and undissolved materials, followed by concentration through a Vivaflow ultrafiltration membrane (Vivascience, Hannover, Germany) with a molecular weight cut-off of 5 kDa. The crude enzyme was loaded onto a FPLC HiTrap Q Sepharose XL 5 ml column (GE Healthcare, Uppsala, Sweden) that was equilibrated with 20 mM Tris–HCl (pH 8.0). Proteins were eluted using a linear gradient of NaCl (0–1.0 M) in the buffer mentioned above at a flow rate of 3.0 ml/min. Fractions exhibiting β-glucosidase activities were pooled and subjected to sodium dodecyl sulfate–polyacrylamide gel electrophoresis (SDS-PAGE). The protein concentration was determined by a Bradford assay with bovine serine albumin as a standard.

### Enzyme expression and purification in *E. coli*

Both the cDNA fragment without the signal peptide-coding sequence and pET-30a(+) were digested by *Nde*I and *EcoR*I and ligated to constructed recombinant plasmids and individually transformed into *E. coli* BL21 (DE3) competent cells. Positive transformants were verified by DNA sequencing. After induction at 30 °C for 6 h by 0.6 mM of isopropyl β-d-1-thiogalactopyranoside (IPTG), the cells (approximately 5 g) were collected by centrifugation at 12,000×*g*, 4 °C for 5 min and resuspended in 25 ml of lysis buffer (20 mM Tris–HCl, pH 7.0), followed by sonication with an Ultrasonic Cell Disruptor (Scientz, China) on ice (5 s short bursts at 200 W followed by an interval of 3 s cooling for 100 times). Cell debris was removed by centrifugation, and the supernatants were subjected to Ni^2+^-NTA chromatography with a linear gradient of imidazole (2–300 mM) in 50 mM Tris–HCl, 0.5 M NaCl, pH 7.6. Fractions exhibiting β-glucosidase activities were pooled and subjected to SDS-PAGE analysis. Elution peaks of a single band with objective size were recovered and concentrated as purified enzyme solution. The protein concentration was determined as described above.

### Enzyme activity assay

The β-glucosidase activity was assayed using *p*NPG or cellobiose as the substrate. One unit of β-glucosidase activity was defined as the amount of enzyme that released 1 μmol of products per minute under the assay conditions. For substrate *p*NPG, the standard reaction system consisted of 250 μl of appropriately diluted enzyme and 250 μl of McIlvaine buffer containing 2 mM *p*NPG. After incubation at a certain temperature for 10 min, 1.5 ml of 1.0 M Na_2_CO_3_ was added into the system to terminate the reaction. The amount of *p*-nitrophenol (*p*NP) released was determined spectrophotometrically by reading the absorbance at 405 nm. Each experiment was performed in triplicate. And for cellobiose, the standard reaction was carried out with 70 μl of appropriately diluted enzyme and 70 μl of McIlvaine buffer containing 2 mM cellobiose for 10 min followed by a boiling water bath to terminate the reaction. GOD-POD coloring solution (2.1 ml) was then added into the system, and the absorbance at 520 nm was determined to calculate the amount of released glucose.

### Biochemical characterization

*p*NPG was used as the substrate for activity assays to investigate the biochemical properties of Bgl3A. The optimal pH for the β-glucosidase activity of purified recombinant Bgl3A was determined at 75 °C for 10 min over a pH range of 2.0–11.0. Buffers used for the assays were as follows: 100 mM Na_2_HPO_4_-citric acid (pH 2.0–8.0), 100 mM Tris–HCl (pH 8.0–9.0), and 100 mM glycine-NaOH (pH 9.0–11.0). To estimate pH stability, the enzyme was pre-incubated in the buffers mentioned above without substrate at 37 °C for 1 h, and the residual activities were measured under the standard conditions (pH 4.5, 75 °C, and 10 min).

The optimal temperature was examined at the pH optimum by measuring the enzyme activity over the temperature range of 30 and 90 °C (pH 4.5 and 10 min). The thermostability was investigated by determining the residual enzyme activities under standard conditions (pH 4.5, 75 °C, and 10 min) after preincubation at 60, 70 and 80 °C and optimal pH without substrate for various periods (10–60 min).

The β-glucosidase activity of purified recombinant Bgl3A was also measured in the presence of 5 mM of various metal ions and chemical reagents (Ag^+^, Ca^2+^, Li^+^, Co^2+^, Cr^3+^, Ni^2+^, Cu^2+^, Mg^2+^, Fe^3+^, Mn^2+^, Hg^2+^, Pb^2+^, EDTA, SDS or β-mercaptoethanol) to estimate their effects. The reaction without any additive was used as a blank control.

### Substrate specificity and kinetic parameters

To investigate the substrate specificity of purified recombinant Bgl3A, 1 % (w/v) of polysaccharides (barley β-glucan, CMC-Na, Avicel, laminarin, lichenin), disaccharides (cellobiose, sophorose, and gentiobiose), soybean flavones (daidzin, genistin, and glycitin) or 1 mM of *p*-nitrophenyl derivatives (*p*NPG, *p*NPAf, *p*NPX, *p*NPGal, *p*NPAb, *p*NPC) were used as the substrate to determine their corresponding activities.

The *K*_m_, *V*_max_ and *k*_cat_ values of purified recombinant Bgl3A were determined under optimal conditions for 5 min in 100 mM Na_2_HPO_4_-citric acid containing 1–10 mM *p*NPG or cellobiose as the substrate. The data were plotted according to the Lineweaver–Burk method.

### Deglycosylation of recombinant Bgl3A produced in *P. pastoris*

To remove *N*-glycans attached during heterologous expression in *P. pastoris*, 5 ml of purified recombinant Bgl3A was incubated with 5000 U (10 µl) of peptide-*N*-glycosidase F (PNGase F, P0704S, New England Biolabs, Ipswich, MA) at 37 °C for 12 h according to the manufacturer’s instructions. To remove *O*-glycans, 4 ml of PNGase F-treated Bgl3A was incubated with 10 µl of α-mannosidase (M7257, Sigma-Aldrich) for 24 h, followed by purification through anion exchange chromatography. Both *N*- and *N*-/*O*-deglycosylated enzyme samples were analyzed by SDS–PAGE.

### Mutant construction and CD analysis

Based on sequence alignment with the close homolog NfBGL1 from *N. fischeri* P1 [43] (73 % identity), a region (residues 417–429) with several potential *O*-glycosylation sites was identified. By replacing the potential *O*-glycosylation sites with corresponding residues of NfBGL1 or alanine with specific primers M1-F/R and M2-F/R (shown in Additional file 1: Table S1), two mutants, i.e., M1 and M2, were constructed to enhance the pH stability of Bgl3A. Enzyme expression and purification of the mutants were conducted as described above.

Far-UV circular dichroism (CD) spectroscopy was used to determine the secondary structures of Bgl3A and its mutants. The spectra between 200 and 260 nm were collected at a protein concentration of 1 mg/ml in PBS buffer (8 g/l NaCl, 1.44 g/l Na_2_HPO_4_, 240 mg/l KH_2_PO_4_, 200 mg/l KCl, pH 7.2) at 25 °C using a MOS-450 CD spectrometer (Bio-Logic, France), which was equipped with a 1-mm path length quartz cuvette and a temperature control device. Each spectrum was an average of three consecutive scans and was corrected by subtracting the buffer spectrum.

### Enzymatic saccharification

Corn stover was pretreated by 1 % NaOH at 121 °C for 30 min in an autoclaver, washed with ddH_2_O, oven-dried, and mixed with 20 ml of 100 mM Na_2_HPO_4_-citric acid buffer (pH 5.0) in 50 ml shake flasks at the concentration of 2 % (dry material). The enzyme(s), i.e., Celluclast 1.5L (Cellulase from *Trichoderma reesei* ATCC 26921, C2730, Sigma-Aldrich) alone (5 FPU/g dry matter) or supplemented with identical units of purified wild type Bgl3A, mutant M1 or commercial liquid Novozyme 188 (Cellobiase from *Aspergillus niger*, C6105, Sigma-Aldrich) (10 BGU/g dry material), respectively, were added into the cellulosic preparations and incubated at 50 °C with an agitation rate of 200 rpm for 96 h. Hydrolyzates were collected at several intervals and centrifuged at 12,000 rpm, 4 °C for 10 min. The amounts of reducing sugars and glucose released in the supernatants were determined using the DNS [62] and GOD-POD methods, respectively. All these experiments were conducted with three replicates.

### Nucleotide sequence accession number

The cDNA sequence of *bgl3A* has been submitted to GenBank under the accession number of KU363626.

## Additional files

10.1186/s13068-016-0560-8 Effect of metal ions and chemical reagents on the activity of purified recombinant β-glucosidase Bgl3A.
10.1186/s13068-016-0560-8 Circular dichroism spectrums of the wild type and mutant proteins of Bgl3A (0.5 mg/ml).
10.1186/s13068-016-0560-8 Primers used in this study.

## Abbreviations

EG: endo-β-glucanase
CBH: cellobiohydrolase
GH: glycosyl hydrolase
PDB: protein data base
*K*_m_: Michaelis constant
*V*_max_: maximum reaction rate
*k*_cat_: turnover number
*p*NP: 4-nitrophenyl
TAIL-PCR: thermal asymmetric interlaced-PCR
SDS-PAGE: sodium dodecyl sulfate–polyacrylamide gel electrophoresis
DNS: 3,5-dinitrosalicylic acid
GOD-POD: glucose oxidase and peroxidase
CD: circular dichroism
