# Supplementary material for: Engineering a highly active thermophilic β-glucosidase to enhance its pH stability and saccharification performance
Source: Biotechnol Biofuels. 2016 Jul 20;9:147. doi: 10.1186/s13068-016-0560-8 (PMC4955127; doi:10.1186/s13068-016-0560-8)
Supplement: Supplementary file 1 — 10.1186/s13068-016-0560-8 Effect of metal ions and chemical reagents on the activity of purified recombinant β-glucosidase Bgl3A. [file 13068_2016_560_MOESM1_ESM.docx]

**Engineering a highly active thermophilic β-glucosidase to enhance its pH stability and saccharification performance**

Wei Xia^1,2^ ^§^, Xinxin Xu^3^ ^§^, Lichun Qian^2^, Pengjun Shi^1^*, Yingguo Bai^1^, Huiying Luo^1^, Rui Ma^1^, Bin Yao^1*^

^1^ Key Laboratory for Feed Biotechnology of the Ministry of Agriculture, Feed Research Institute, Chinese Academy of Agricultural Sciences, Beijing 100081, People’s Republic of China

^2^ College of Animal Science, Zhejiang University, Hangzhou 310058, People’s Republic of China

^3^ Biotechnology Research Institute, Chinese Academy of Agricultural Sciences, Beijing 100081, People’s Republic of China.

^§^W. X. and X. X. contributed equally to this paper.

^*^Corresponding author to Pengjun Shi and Bin Yao, Key Laboratory for Feed Biotechnology of the Ministry of Agriculture, Feed Research Institute, Chinese Academy of Agricultural Sciences, No. 12 Zhongguancun South Street, Beijing 100081, People’s Republic of China. Phone: 86-10-82106053. Fax: 86-10-82106054.

E-mail: [shipengjun@caas.cn](mailto:shipengjun@caas.cn); yaobin@caas.cn.

**Additional file 1.** Effect of metal ions and chemical reagents on the activity of purified recombinant β-glucosidase Bgl3A.

| Chemicals | Relative activity (%) ^a^ | Chemicals | Relative activity (%) ^a^ | |
| --- | --- | --- | --- | --- |
| None | 100.0 ± 0.9 | Fe^2+^ | | 90.7 ± 2.3 |
| Ca^2+^ | 120.8 ± 4.7 | Zn^2+^ | | 78.0 ± 3.1 |
| Mg^2+^ | 102.8 ± 0.9 | Cu^2+^ | | 42.0 ± 1.8 |
| Mn^2+^ | 102.4 ± 2.1 | Ag^+^ | | 12.4 ± 1.5 |
| Co^2+^ | 99.6 ± 1.9 | EDTA | | 83.0 ± 2.5 |
| Cr^3+^ | 99.3 ± 2.6 | SDS | | 78.5 ± 2.5 |
| Pb^2+^ | 94.2 ± 0.6 | β-Mercaptoethanol | | 70.0 ± 2.1 |
| Ni^2+^ | 92.1 ± 0.7 |  | |  |

^a^ Relative activity is defined as the percentage of enzymatic activity in the presence of chemicals against that without any addition and is shown as mean ± standard deviation (n = 3).

**Additional file 2.** Circular dichroism spectrums of the wild type and mutant proteins of Bgl3A (0.5 mg/ml).


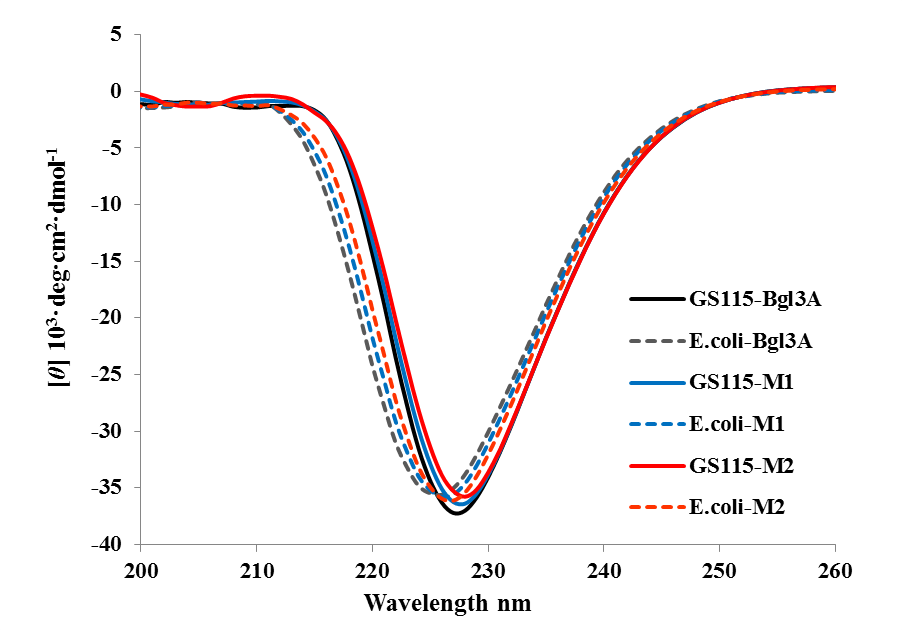


**Additional file 3.** Primers used in this study.

| Primer name | Primer sequence (5′→3′) ^a^ |
| --- | --- |
| DP-F | GGCCGCAAYTGGGARGGNTT |
| DP-R | GTCACCAGGCATNGHCATRTC |
| usp1 | GTAGAGCTTGGCCTTAGGGTCGAC |
| usp2 | GCGGCGGTCTCGAAGGCAAGCTTG |
| usp3 | GGTAGGTTCCGTCCTCGTTCAGCG |
| dsp1 | CGAGGACGGAACCTACCGCGAGAGC |
| dsp2 | CGAGGAGTACATCAAGCTTGCCTTCG |
| dsp3 | CAAGGTCGACCCTAAGGCCAAGC |
| GS115-PF | GGGGAATTCTATGGCTTCGGCGGCTCTGGCTG |
| GS115-PR | GGGGCGGCCGCTCAAATACGGAAAGATTCCTGCT |
| *E. coli*-PF | GGGCATATGCACCATCACCATCACCATTATGGCTTCGGCGGCTCTGGCTG |
| *E. coli*-PR | GGGGAATTCTCAAATACGGAAAGATTCCTGCT |
| M1-F | GTCCCCAGCACCACCGACGACCCCACCGCGGCAGCTTCTGCAGCCG |
| M1-R | GGGGTCGTCGGTGGTGCTGGGGACGATGTTAGTGCCATCAGCGGCAGC |
| M2-F | CTGATGGCACTAACATCGCCGCCAGCACGACCGATGATGCCACCGCGGC |
| M2-R | GCAGAAGCTGCCGCGGTGGCATCATCGGTCGTGCTGGCGGCGATGTTAG |

^a^ The restriction sites are underlined, and the coding sequence of His_6_ tag is framed.
