# Supplementary material for: Engineering a highly active thermophilic β-glucosidase to enhance its pH stability and saccharification performance
Source: Biotechnol Biofuels. 2016 Jul 20;9:147. doi: 10.1186/s13068-016-0560-8 (PMC4955127; doi:10.1186/s13068-016-0560-8)

**Additional file 2.** Circular dichroism spectrums of the wild type and mutant proteins of Bgl3A (0.5 mg/ml).


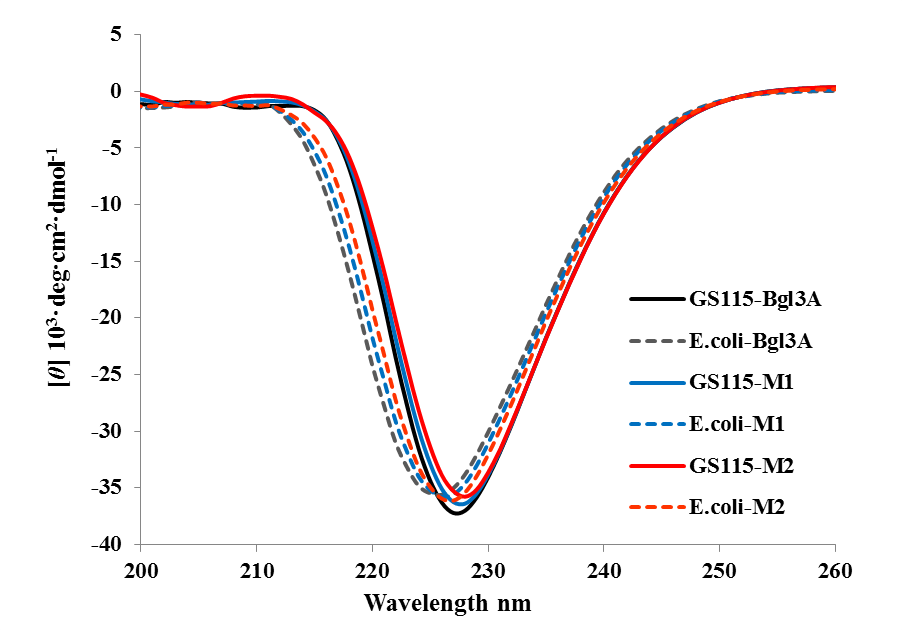

Supplement: Supplementary file 2 — 10.1186/s13068-016-0560-8 Circular dichroism spectrums of the wild type and mutant proteins of Bgl3A (0.5 mg/ml). [file 13068_2016_560_MOESM2_ESM.docx]
