# Supplementary material for: Engineering a highly active thermophilic β-glucosidase to enhance its pH stability and saccharification performance
Source: Biotechnol Biofuels. 2016 Jul 20;9:147. doi: 10.1186/s13068-016-0560-8 (PMC4955127; doi:10.1186/s13068-016-0560-8)
Supplement: Supplementary file 3 — 10.1186/s13068-016-0560-8 Primers used in this study. [file 13068_2016_560_MOESM3_ESM.docx]

**Additional file 3.** Primers used in this study.

| Primer name | Primer sequence (5′→3′) ^a^ |
| --- | --- |
| DP-F | GGCCGCAAYTGGGARGGNTT |
| DP-R | GTCACCAGGCATNGHCATRTC |
| usp1 | GTAGAGCTTGGCCTTAGGGTCGAC |
| usp2 | GCGGCGGTCTCGAAGGCAAGCTTG |
| usp3 | GGTAGGTTCCGTCCTCGTTCAGCG |
| dsp1 | CGAGGACGGAACCTACCGCGAGAGC |
| dsp2 | CGAGGAGTACATCAAGCTTGCCTTCG |
| dsp3 | CAAGGTCGACCCTAAGGCCAAGC |
| GS115-PF | GGGGAATTCTATGGCTTCGGCGGCTCTGGCTG |
| GS115-PR | GGGGCGGCCGCTCAAATACGGAAAGATTCCTGCT |
| *E. coli*-PF | GGGCATATGCACCATCACCATCACCATTATGGCTTCGGCGGCTCTGGCTG |
| *E. coli*-PR | GGGGAATTCTCAAATACGGAAAGATTCCTGCT |
| M1-F | GTCCCCAGCACCACCGACGACCCCACCGCGGCAGCTTCTGCAGCCG |
| M1-R | GGGGTCGTCGGTGGTGCTGGGGACGATGTTAGTGCCATCAGCGGCAGC |
| M2-F | CTGATGGCACTAACATCGCCGCCAGCACGACCGATGATGCCACCGCGGC |
| M2-R | GCAGAAGCTGCCGCGGTGGCATCATCGGTCGTGCTGGCGGCGATGTTAG |

^a^ The restriction sites are underlined, and the coding sequence of His_6_ tag is framed.
